# Lithobezoar and Phytobezoar Causing Intestinal Obstruction: A Report of Two Cases

**DOI:** 10.1002/ccr3.70293

**Published:** 2025-03-02

**Authors:** Bishnu Prasad Kandel, Anup Chalise, Sujan Shrestha, Paleswan Joshi Lakhey

**Affiliations:** ^1^ Department of Surgical Gastroenterology Tribhuvan University Teaching Hospital Kathmandu Nepal; ^2^ Department of Surgery North Middlesex University Hospital London UK; ^3^ Department of GI and General Surgery Manipal Teaching Hospital Pokhara Nepal

**Keywords:** bezoar, gastric outlet obstruction, intestinal obstruction, lithobezoar, phytobezoar, stricture

## Abstract

Concretion or mass formed of exogenous undigested material in the gastrointestinal tract is called bezoar. Bezoar is a rare condition and can present with clinical features ranging from recurrent abdominal pain to acute presentation with obstruction or gastrointestinal bleeding. Preoperative diagnosis is usually done by imaging studies. They are treated with endoscopic or surgical removal of the bezoar along with treatment of complications and underlying illness. Here, we present two cases of bezoars: first, a case of a duodenal lithobezoar in 35‐year‐old male who presented with features of gastric outlet obstruction. He was found to have duodenal stricture and multiple small lithobezoars in the stomach and duodenum. The bezoars were removed by laparotomy and gastrotomy; and gastrojejunostomy was done to bypass the stricture. The second was a jejunal phytobezoar in 42‐year‐old male who presented with jejunal obstruction. Laparotomy and resection of the involved segment of jejunum and end‐to‐end anastomosis were done. Both the patients improved without postoperative complications.

## Graphical abstract

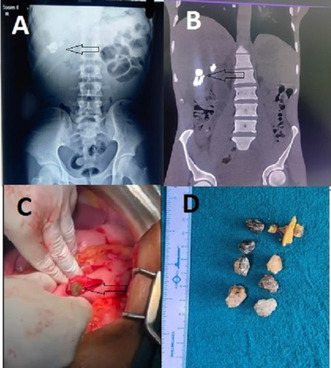

**Summary**

- Bezoars are rare causes of abdominal pain and intestinal obstruction.
- Gastric or small intestinal bezoars often need surgical management.
- Good outcomes can be expected after surgery for small intestinal bezoars.

## 1 Introduction

Bezoar is a concretion or mass formed of exogenous undigested material in the gastrointestinal tract. Gastrointestinal bezoars are rare conditions with various case reports and case series reported in the literature. In a report of endoscopic diagnosis of gastric bezoar, 49 cases were detected in 20 years period, accounting for 0.068% of all ensocopies [1]. Ghosheh et al. [2] analyzed 1061 cases of acute intestinal obstruction and found bezoars as the cause of obstruction in 0.8% of cases. Although bezoars are commonly found in the stomach, they can be found anywhere in the gastrointestinal tract [3]. Patients with altered gastrointestinal anatomy or motility, poor mastication, excessive intake of fibers, or psychiatric illness are at risk for the development of bezoars [4]. Based on the composition, different types of bezoars are phytobezoars (composed of plant fibers), trichobezoars (composed of a conglomeration of hair and food particles), lactobezoars (composed of milk protein), lithobezoar (composed of stones or soil) or pharmacobezoars (which are concretions of various medications) [5]. Bezoars do not have specific symptoms or signs, and most of them present with nonspecific pain in the abdomen or features of intestinal obstruction. Upper gastrointestinal endoscopy can easily detect the gastric bezoar, whereas computed tomography (CT) scans have been found to be a reliable and accurate tool to identify the presence of gastrointestinal (GI) bezoar [6, 7]. Endoscopic removal of the bezoars can be possible in selected cases, but most of them require surgical management.

## 2 Case Presentation

### 2.1 Case Number One

#### 2.1.1 Case History/Examination

We present cases of a 35‐year‐old male who presented to our hospital with complaints of recurrent upper abdominal pain and non‐bilious vomiting for 2 months. He was diagnosed with mild intellectual developmental disorder but was not on regular follow‐up or any medications. There was no history of other significant illness in the past. Examination findings were normal except for fullness in the upper abdomen.

#### 2.1.2 Methods (Differential Diagnosis, Investigations and Treatment)

**FIGURE 1:**
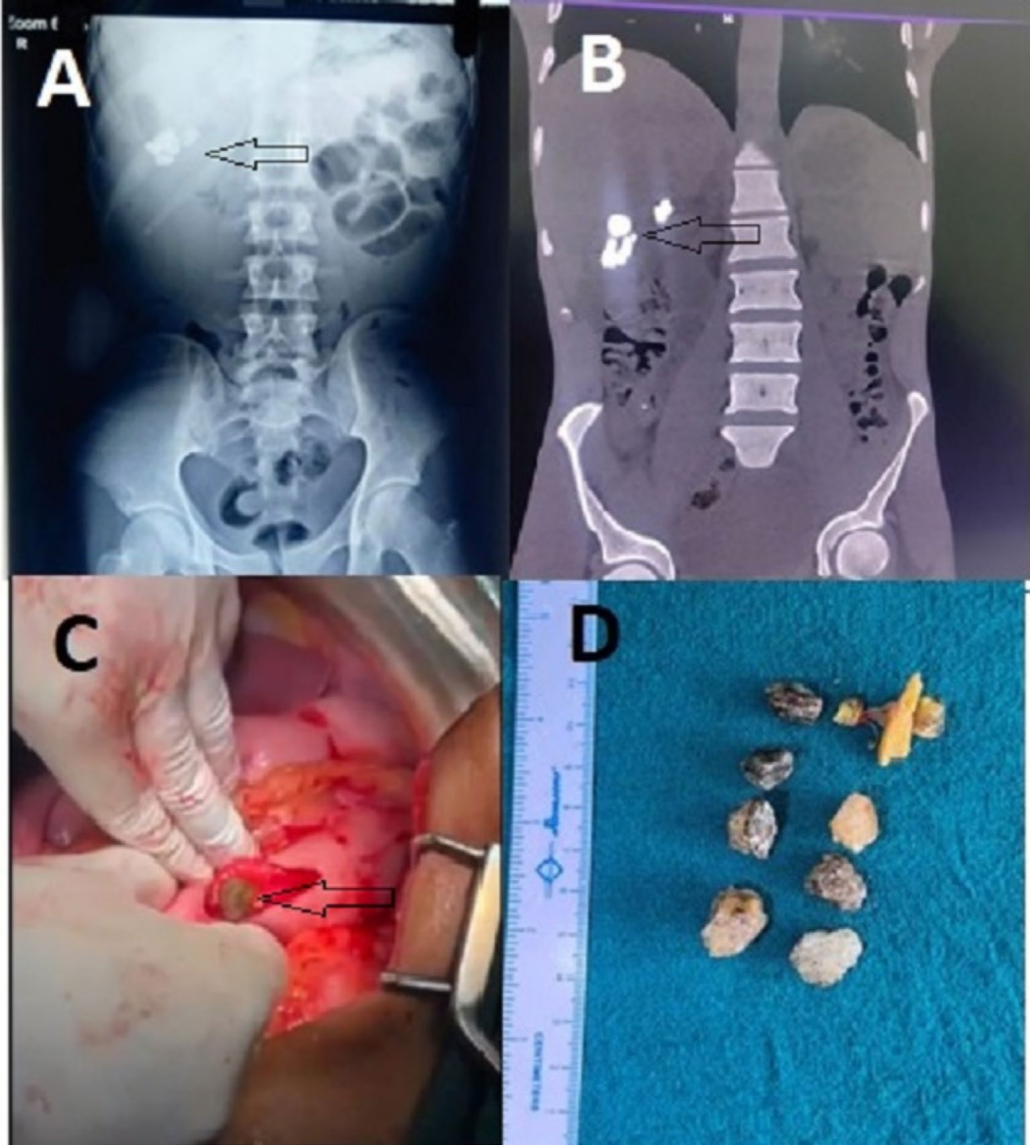
Imges of case 1; (A) X‐ray abdomen showing radiopaque findings in duodenum (Arrow); (B) CT scan of abdomen showing multiple radio opaque material in duodenum (Arrow); (C) Gastrotomy with removal of lithobezoar (Arrow); (D) Multiple lithobezoar removed from the duodenum.

Routine investigations including complete blood counts, blood sugar, blood urea nitrogen, and liver function tests were normal. Xray of the abdomen revealed multiple hyperdense structures at the right upper quadrant of the abdomen and CT scan shows similar multiple hyperdense structures in the first part of the duodenum (Figure 1A,B). Upper gastrointestinal endoscopy was done after nasogastric tube placement and lavage, which revealed a stricture at the junction of the first and second parts of the duodenum and multiple foreign bodies above the stricture, which was attempted to be removed endoscopically but failed. Since the patient had features of gastric outlet obstruction with a benign duodenal stricture and multiple foreign bodies proximal to the stricture, he was planned for surgical extraction of the foreign bodies and gastrojejunostomy.

During laparotomy, there was a distended stomach and the first part of the duodenum with palpable foreign bodies in the first part of the duodenum. The foreign bodies in the duodenum were carefully milked to the stomach. A gastrotomy of about 3 cm was made on the anterior wall of the distal stomach, and the foreign bodies were extracted from the gastrotomy site (Figure 1C). To our utter surprise, multiple pebbles were retrieved as foreign bodies (seven in number) and one wooden piece was retrieved (Figure 1D). Gastrojejunostomy was done at the same gastrotomy site with a loop of jejunum 40 cm distal to the duodenojejunal flexure to bypass the duodenal stricture. The patient had a normal postoperative course, and an oral diet was started on postoperative day two. He was discharged on postoperative day seven without any surgical complications.

### 2.2 Case Number Two

#### 2.2.1 Case History/Examination

We present a case of 42‐year‐old male with a history of colic‐type upper abdominal pain and bilious vomiting for 3 days. He had a history of a similar episode 6 months back, which resolved after conservative management. Examination findings were unremarkable except for mild abdominal fullness.

#### 2.2.2 Methods (Differential Diagnosis, Investigations and Treatment)

**FIGURE 2:**
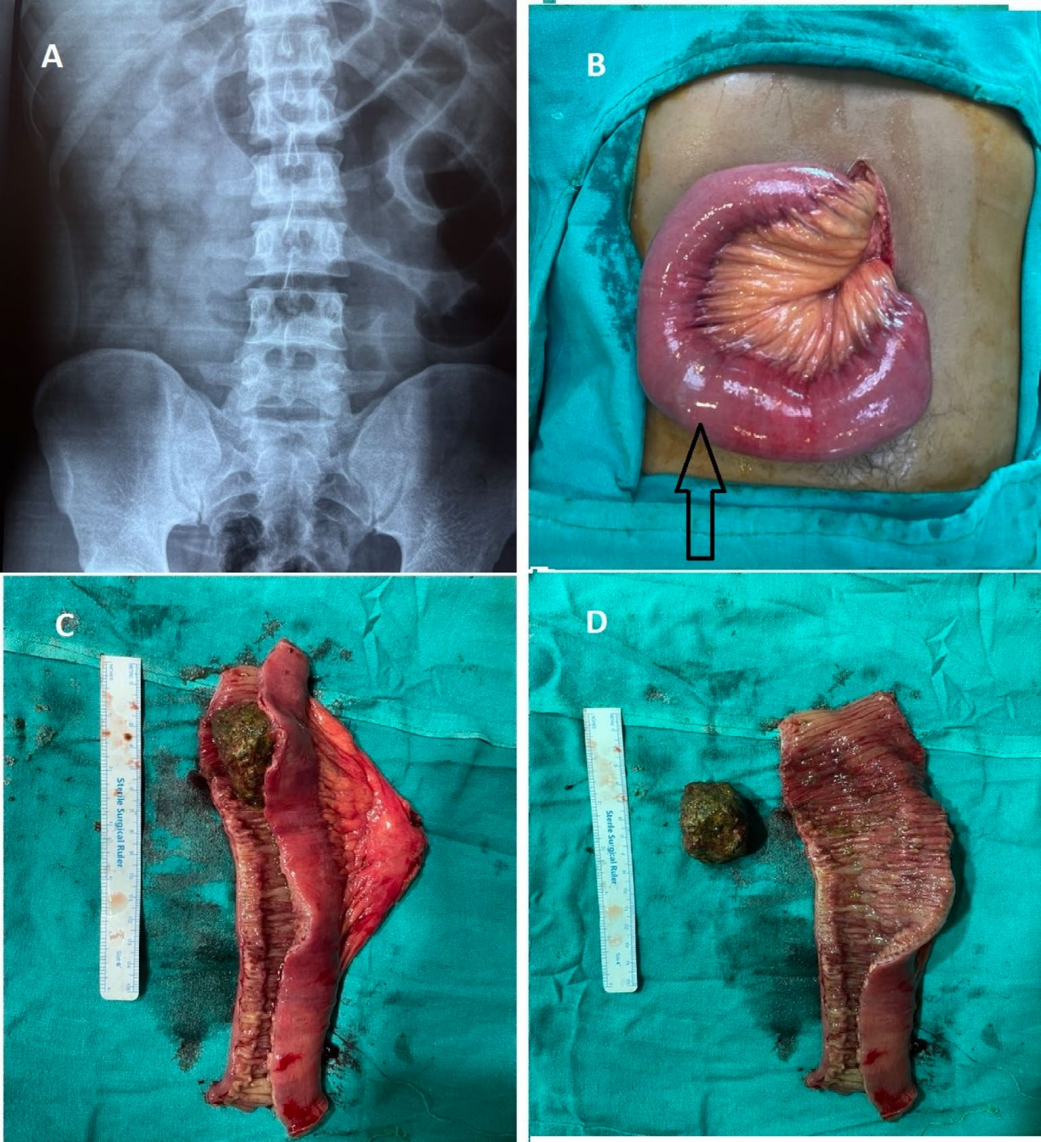
Images of case 2. (A) X‐ray abdomen showing features of jejunal obstruction; (B) intraoperative picture of dilated jejunum containing phytobezoar (Arrow); (C) Resected jejunum; (D) Phytobezoar and the jejunal mucosa.

Routine investigations, including complete blood counts, renal function tests, and liver function tests, were within normal limits. X‐ray abdomen erect and supine views show features of small intestine obstruction with multiple air‐fluid levels (Figure 2A). The patient did not improve with conservative management with intravenous fluids, analgesics, and antiemetic medications, and exploratory laparotomy was performed after 48 h. Intraoperative findings included a dilated distended jejunum with a mass of about 7 cm felt inside the jejunum 200 cm distal to dudenojejunal flexure (Figure 2B). Resection of the jejunum with 10 cm margins from the mass and end‐to‐end anastomosis was performed. When the resected jejunum was opened, there was a 5‐cm foreign body consisting of undigested vegetable fibers clumped together, causing the intestinal obstruction (Figure 2C,D). The postoperative period was uneventful, and the patient was discharged on the sixth postoperative day.

## 3 Conclusion and Results (Outcome and Follow‐Up)

Both of the patients improved after surgery without any complications. The first patient was asymptomatic on follow‐up at 9 months after surgery and was on regular follow‐up with the psychiatrist. The second patient was also asymptomatic at 6 months follow‐up after the surgery. X‐ray abdomen was done for both patients at 6 months after surgery, which showed normal findings.

## 4 Discussion

The term “bezoar” is thought to be derived from the Arabic word “badzehr” or the Persian word “panzehr,” both of which mean “counterpoison,” or “antidote” [8]. The presentation of bezoar depends upon its composition. Lactobezoars may present in premature infants or newborns with symptoms of feeding intolerance and abdominal distension. Trichobezoar and phytobezoar usually present with upper abdominal pain and features of gastric outlet obstruction. Lithobezoars are more common in the large intestine and present with features of colonic obstruction [9]. Seed bezoars are most commonly found in the rectum and terminal ileum, and most cases of seed bezoar are reported from Middle Eastern countries [10]. Our first patient presented with features of gastric outlet obstruction, and it was attributed to benign duodenal stricture rather than to lithobezoar. The second patient presented with features of small intestine obstruction, and diagnosis was made after resection of the jejunal segment.

Gastric bezoars can be removed or fragmented by endoscopy [11, 12] whereas bezoars in the rectum can be removed manually. Endoscopic management was not successful in our patient. Medical management by enzymatic degradation can be possible for phytobezoars. Dissolution of gastric phytobezoars by nasogastric lavage with cellulose has been reported [5]. Acetylcysteine or commercially available soft drinks have been used with various success rates for the dissolution of lactobezoar [13, 14]. Surgical removal is required for most tricobezoars or any bezoar with complications such as intestinal obstruction, perforation, or bleeding. Both of our patients had intestinal obstruction and were managed surgically. These cases highlight a rare instance of intestinal obstruction due to lithobezoar and phytobezoar. Successful management through elective surgery emphasizes the importance of tailored interventions in these cases.

## Author Contributions

**Bishnu Prasad Kandel:** conceptualization, data curation, formal analysis, methodology, writing – original draft, writing – review and editing. **Sujan Shrestha:** data curation, investigation, methodology, resources, writing – review and editing. **Anup Chalise:** formal analysis, investigation, project administration, resources, writing – original draft. **Paleswan Joshi Lakhey:** methodology, supervision, visualization, writing – review and editing.

## Consent

Written informed consent was obtained from the patients for the publication of this case report.

## Conflicts of Interest

The authors declare no conflicts of interest.

## Data Availability

Data are available on request.
